# Effect of MP‐AzeFlu compared to monotherapy on COX‐2, PGE_2_, and EP2 gene expression in upper airway mucosa

**DOI:** 10.1002/iid3.709

**Published:** 2022-12-15

**Authors:** Sonia Vicens‐Artes, Jordi Roca‐Ferrer, Valeria Tubita, Mireya Fuentes, Isam Alobid, Antonio Valero, Ferdinand Kopietz, DucTung Nguyen, Joaquim Mullol

**Affiliations:** ^1^ Clinical and Experimental Respiratory Immunoallergy, IDIBAPS Barcelona Spain; ^2^ CIBER of Respiratory Diseases (CIBERES) Barcelona Spain; ^3^ Clinical and Experimental Respiratory Immunoallergy Universitat de Barcelona Barcelona Spain; ^4^ Rhinology Unit & Smell Clinic, ENT Department Hospital Clinic Barcelona Barcelona Spain; ^5^ Allergy Section Pulmonology & Allergy Department Barcelona Spain; ^6^ MEDA Pharma GmbH & Co. KG (A Viatris Company) Bad Homburg Germany

**Keywords:** COX‐2, EP2, fluticasone, MP‐AzeFlu, PGE_2_

## Abstract

MP‐AzeFlu (intranasal fluticasone and azelastine) has been widely studied and has demonstrated efficacy in Allergic rhinitis with a superior effect compared to these drugs administered individually; however, the mechanism by which MP‐AzeFlu produces this improved clinical effect has not yet been fully explained. In this study, we investigated the effect of MP‐AzeFlu and fluticasone propionate (FP) on arachidonic acid metabolism as measured by changes in regulation of cyclooxygenase (COX) isoforms, prostaglandin (PG) D_2_, PGE_2_, PGE_2_ receptor (EP) 2, and EP3. Expression of these key inflammation markers was assessed through an in vitro model of upper airway inflammation using fibroblasts derived from both healthy and inflamed upper airway mucosa. Both MP‐AzeFlu and FP inhibited interleukin‐1β‐induced COX‐2 messenger RNA (mRNA) and protein expression and PGE_2_ secretion in vitro. MP‐AzeFlu and FP both upregulated EP2 mRNA expression, though neither upregulated EP2 protein expression. This downregulation of COX‐2 and PGE_2_ coupled with upregulation of EP2 receptor expression reinforces the anti‐inflammatory effect of MP‐AzeFlu in upper airway inflammation.

## Graphical abstract

In this study, we investigated the effect of intranasal fluticasone and azelastine (MP‐AzeFlu) and fluticasone propionate (FP) on arachidonic acid metabolism as measured by changes in regulation of cyclooxygenase (COX) isoforms, prostaglandin (PG) D2, PGE2, PGE2 receptor (EP) 2, and EP3. Both MP‐AzeFlu and FP inhibited interleukin‐1β‐induced COX‐2 messenger RNA (mRNA) and protein expression and PGE2 secretion in vitro. MP‐AzeFlu and FP both upregulated EP2 mRNA expression, though neither upregulated EP2 protein expression.

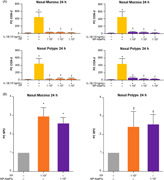

1

To the Editor:

Allergic rhinitis (AR) is a disease caused by IgE‐mediated reactions that increase cell expression of t‐helper 2 (Th2) cytokines (type 2 inflammation) and lead to infiltration of eosinophils into nasal tissue and secretion of mucus.^1^ MP‐AzeFlu (intranasal fluticasone and azelastine) has been widely studied and has been shown to reduce inflammatory mediators and nasal hyperreactivity.^2^ Additionally, MP‐AzeFlu has demonstrated efficacy in AR with a superior effect compared to its component drugs administered individually.^3^, ^4^, ^5^ On the other hand, the mechanisms by which this medication improves symptoms of AR have not been fully elucidated.

While the role of epithelial cells on AR pathogenesis has been widely studied, there is increasing evidence that fibroblasts also play a prominent role in AR. Several studies have demonstrated that fibroblasts release proinflammatory mediators involved in the molecular mechanisms present in the airways of patients suffering from AR. For example, primary nasal fibroblasts isolated from patients with AR showed higher proliferation and migration abilities and increased expression of interleukin (IL)‐33 and IL‐6 compared to controls.^6^ Furthermore, it has been demonstrated that human fibroblasts from patients with AR release thymic stromal lymphopoietin (TSLP) in response to IL‐17A. Since IL‐17A has been implicated in the pathogenesis of AR and TSLP modifies the immune response toward a Th2 phenotype, these results suggest that fibroblasts play a role in the development of AR.^7^ Finally, human nasal fibroblasts showed increased expression of IL‐6, IL‐1β, and TNF‐α in response to urban particulate matter, indicating a relationship between fibroblasts and AR pathogenesis.^8^ Consequently, upper airway fibroblasts represent a reliable in vitro model to assess the mechanism involved in these inflammatory diseases due to the relevance of these structural cells in the pathogenesis of AR.^9^

Our laboratory has previously developed in vitro models to study the etiology of upper airways inflammatory diseases as well as the effect and potency of anti‐inflammatory drugs.^10^, ^11^ We have reported that abnormalities in arachidonic acid (AA) metabolism are present in structural cells of upper airways inflammatory diseases.^10^ In fact, we demonstrated alterations in the regulation of AA metabolism in cultured fibroblasts using IL‐1β as a proinflammatory stimuli.^10^, ^11^

The objective of this study was to assess the effect of MP‐AzeFlu and fluticasone propionate (FP) on AA metabolism by measuring the expression of cyclooxygenase (COX) isoforms, prostaglandin (PG) D_2_, PGE_2_, PGE_2_ receptor (EP) 2, and EP3 in cultured fibroblasts from healthy and inflamed upper airway mucosa using an in vitro model of inflammation.

Nasal fibroblasts were obtained from nasal mucosa (NM) and nasal polyp (NP) tissues from patients undergoing endoscopic sinonasal surgery and isolated using a specific and selective growth culture media. The purity of fibroblast cultures was confirmed by positive immunostaining to vimentin and negative to cytokeratin 1. Isolated fibroblasts were cultured in a serum‐supplemented medium as previously described.^10^ Cells were treated for 1 to 24 h with MP‐AzeFlu or FP (dilutions 1:10^2^, 1:10^3^,1:10^4^) with or without 10 ng/ml IL−1β. COX‐2 and EP2 messenger RNA (mRNA) gene and protein expression were assessed, as well as PGE_2_ secretion.

### 1.1 COX‐2 expression

IL‐1β induced COX‐2 mRNA expression at 6 (data not shown) and 24 h (Figure 1, Panel A) in NM and NP. MP‐AzeFlu and FP inhibited IL‐1β‐induced COX‐2 mRNA expression at all dilutions at 6 (*p* < .05; data not shown) and 24 h (*p* < .05; Figure 1, Panel A) in NM and NP. IL‐1β also induced COX‐2 protein expression at 24 h in NM and NP. MP‐AzeFlu and FP inhibited IL‐1β‐induced COX‐2 protein expression at all dilutions at 24 h in NM (*p* < .05) and NP (not significant) (Figure 2, Panel A).

**Figure 1:**
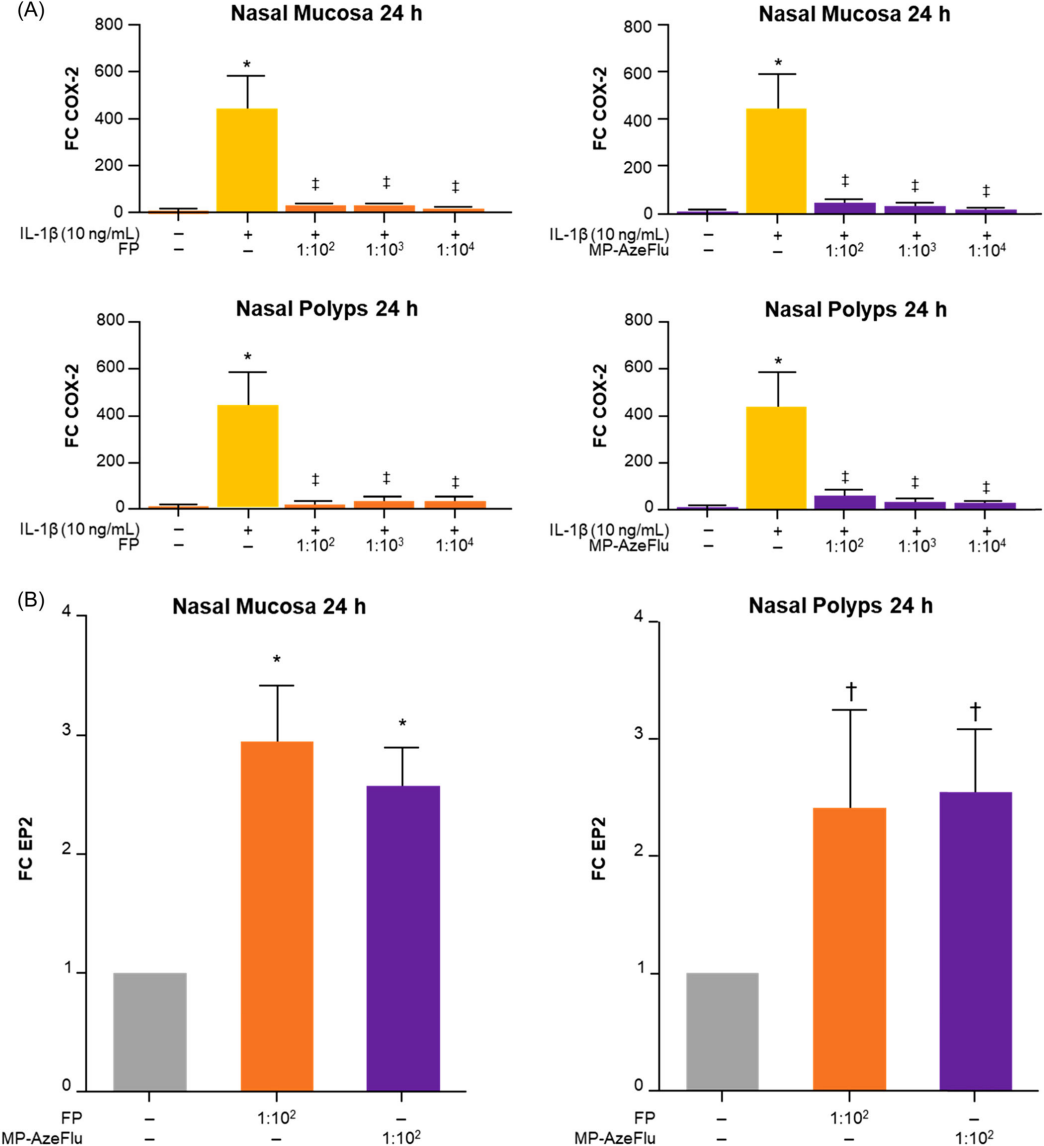
MP‐AzeFlu and fluticasone effects on COX‐2 and EP2 mRNA expression at 24 h. (Panel A) MP‐AzeFlu and FP effect on COX‐2 mRNA expression. (Panel B) MP‐AzeFlu and FP effect on EP2 mRNA expression. *p < .05 compared with culture media alone; ^†^ *p* < .01 compared with culture media alone; ^‡^ *p* < .05 compared with IL‐1β. FC, fold change; FP, fluticasone; IL, interleukin; MP‐AzeFlu, intranasal fluticasone and azelastine; mRNA, messenger RNA.

**Figure 2:**
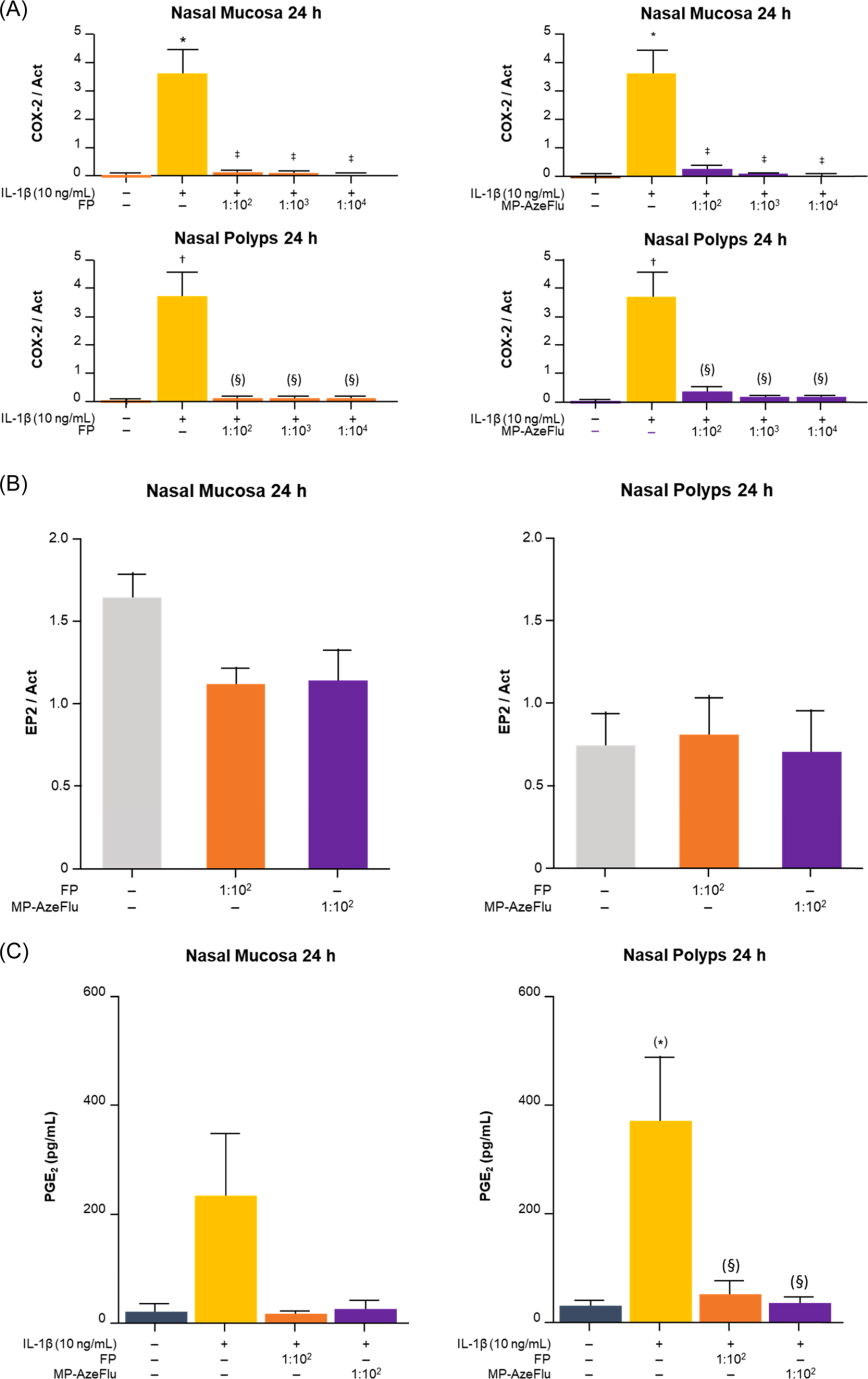
MP‐AzeFlu and fluticasone effects on COX‐2 and EP2 protein expression and PGE_2_ secretion. (Panel A) Effect of MP‐AzeFlu and FP on COX‐2 protein expression. (Panel B) Effect of MP‐AzeFlu and FP on EP2 protein expression. (Panel C) Effect of MP‐AzeFlu and FP on PGE_2_ secretion. *p = .06 compared with culture media alone. *p < .001 compared with culture media alone; ^†^ *p* < .01 compared with culture media alone; ^‡^ *p* < .05 compared with IL‐1β; ^(§)^ *p* < .06 compared with IL‐1β. Act, β‐actin; FP, fluticasone; IL, interleukin; MP‐AzeFlu, intranasal fluticasone and azelastine.

### 1.2 EP2 expression

MP‐AzeFlu and FP upregulated EP2 mRNA expression at dilution 1:10^2^ at 24 h in NM (*p* < .05) and NP (*p* < .01; Figure 1, Panel B). Neither MP‐AzeFlu nor FP upregulated EP2 protein expression at dilution 1:10^2^ in NM or NP (Figure 2, Panel B).

### 1.3 PGE_2_ expression

IL‐1β induced PGE_2_ secretion at 24 h in NM and NP without reaching statistical significance. MP‐AzeFlu and FP demonstrated inhibition of IL‐1β‐induced PGE_2_ secretion at dilution 1:10^2^ at 24 h in NM and NP without reaching statistical significance (Figure 2, Panel C).

### 1.4 COX‐1, EP3, and PGD_2_

IL‐1β did not induce mRNA expression of COX‐1 or EP3 nor protein secretion of PGD_2_ in fibroblasts (data not shown).

In this study, we examined the effects of MP‐AzeFlu and FP on the expression of COX isoforms, PGD_2,_ PGE_2_, EP2, and EP3 in cultured fibroblasts from both healthy and inflamed upper airway mucosa. We demonstrated that both MP‐AzeFlu and FP inhibited IL‐1β‐induced COX‐2 mRNA and protein expression in NM and NP. In addition, MP‐AzeFlu and FP also inhibited IL‐1β‐induced PGE_2_ secretion at dilution 1:10^2^ at 24 h in NM and NP. Finally, MP‐AzeFlu and FP both upregulated EP2 mRNA expression in NM and NP; however, neither upregulated EP2 protein expression. The root cause of the discrepancy between message levels and protein expression is unknown but is likely a result of regulation events occurring between transcription of the mRNA and translation of the protein product. This discordance is frequently described in the literature, as protein products and RNA are single steps in a complex, multi‐step molecular process involving dynamic production, modification, and degradation of messages, intermediates, and products.^12^ Genome‐wide studies of the correspondence between mRNA and protein have shown poor correlation between message and product expression levels.^13^ Finally, a limitation of this study is that the drug concentrations used in this in vitro environment do not correspond to clinical doses and interactions between cells is lost; thus, these findings may not translate to clinical outcomes. In addition, the endotype of chronic rhinosinusitis with nasal polpys (CRSwNP) can be quite complex, and the inflammatory profile of the CRSwNP samples were not obtained. However, because the patient population included in this study was primarily from Spain (a Western country), it can be reasonably assumed that these individuals with CRSwNP would primarily be characterized as having a T2 endotype.^14^, ^15^ In fact, recent data (*Rhinology*, in press) from our research group indicate that 84% of patients with CRSwNP in Spain have a T2 endotype. This rate increases to 87% in patients severely affected by CRSwNP and 91% of those with airway multimorbidities.^16^

In summary, the downregulation of COX‐2 and PGE_2_, together with the upregulation of the EP2 receptor, reinforces the anti‐inflammatory effect of MP‐AzeFlu in upper airway inflammation.

Additional information about study methods and findings are available at https://doi.org/10.5281/zenodo.6038328

## AUTHOR CONTRIBUTIONS

All authors made substantial contributions to the conception or design of the manuscript, or the acquisition, analysis, or interpretation of data for the manuscript, and all authors were involved in drafting the manuscript or revising it critically for important intellectual content. The authors were fully responsible for all content and editorial decisions and received no financial support or other form of compensation related to the development of this manuscript. All authors had final approval of the manuscript and are accountable for all aspects of the work in ensuring the accuracy and integrity of this manuscript.

## CONFLICT OF INTEREST

Joaquim Mullol is or has been a member of national and international scientific advisory boards (consulting), received fees for lectures, and grants for research projects from Allakos, AstraZeneca, Genentech‐Roche, Glenmark, GSK, Menarini, MSD, Mitsubishi‐Tanabe, MYLAN‐MEDA Pharma (Viatris), Novartis, Procter and Gamble, Sanofi‐Genzyme and Regeneron, UCB, and Uriach Group. The remaining authors declare no conflict of interest.

## ETHICS STATEMENT

The Dymecos 2 study was approved by the Ethics Committee (CEIm) from Hospital Clinic Barcelona (Catalonia, Spain) on February 3, 2016 with the Registration N^o^ HCB/2016/0007.

## Data Availability

The data that support the findings of this study are available from the corresponding author upon reasonable request.
